# Early onset severe preeclampsia and eclampsia in a low-resource setting, Mpilo Central Hospital, Bulawayo, Zimbabwe

**DOI:** 10.1186/s13104-019-4865-0

**Published:** 2019-12-21

**Authors:** Solwayo Ngwenya, Brian Jones, Desmond Mwembe, Cladnos Mapfumo, Akinbowale Familusi, Hausitoe Nare, Alexander Edward Patrick Heazell

**Affiliations:** 1 Department of Obstetrics & Gynaecology, Mpilo Central Hospital, Vera Road, Mzilikazi, P.O. Box 2096 Bulawayo, Matabeleland Zimbabwe; 2 Royal Women’s Clinic, 52A Cecil Avenue, Hillside, Bulawayo, Zimbabwe; 3 National University of Science and Technology Medical School, Ascot, P. O. Box AC 939 Bulawayo, Matabeleland Zimbabwe; 4 Tommy’s Research Centre Manchester, Academic Health Science Centre, Faculty of Biology, Medicine and Health, The University of Manchester, 5th Floor (Research), St Mary’s Hospital Oxford Road, Manchester, M13 9WL UK

**Keywords:** Early onset severe preeclampsia, Eclampsia, Incidence, Low-resource setting

## Abstract

**Objectives:**

Early-onset severe preeclampsia is associated with significant maternal and perinatal morbidity and mortality especially in low-resource settings, where women have limited access to antenatal care. This dataset was generated from a retrospective cross-sectional study carried out at Mpilo Central Hospital, covering the period February 1, 2016 to July 30, 2018. The aim of the study was to determine the incidence of early-onset severe preeclampsia and eclampsia, and associated risk factors in a low-resource setting. The reason for examining the incidence of preeclampsia specifically in a low-resource setting; was to document it as women in these settings appear to suffer from poor outcomes.

**Data description:**

The dataset contains data of 238 pregnant women who had a diagnosis of early onset severe preeclampsia/eclampsia. There were 243 babies from singleton and twin gestations. There were five sets of twins. There were 21,505 live births during the study period giving an incidence of 1.1%. The dataset contains data on maternal socio-demographic, signs and symptoms, therapeutic interventions and mode of delivery, adverse outcomes characteristics, and fetal characteristics. This large dataset can be used to calculate the incidence and risk factors for adverse maternal and fetal outcomes or develop predictive models in severe preeclampsia/eclampsia.

## Objectives

Early onset severe preeclampsia and eclampsia are of great interest because they are associated with significant maternal and perinatal morbidity and mortality, especially in low and middle-income countries. This dataset was generated from a retrospective cross-sectional study carried out at Mpilo Central Hospital, covering the period February 1, 2016 to July 30, 2018. Mpilo Central Hospital is located in the township of Mzilikazi in Bulawayo. Bulawayo, located in Matabeleland is the second largest city in Zimbabwe after the capital city Harare. The aim of the study was to determine the incidence of early-onset severe preeclampsia and eclampsia, and associated risk factors in a low-resource setting. Early onset severe preeclampsia was diagnosed in those patients with high blood pressure (systolic blood pressure ≥ 160, diastolic blood pressure ≥ 110 mmHg) and either or severe headaches, epigastric pains and deranged biochemical/haematological blood indices. Eclampsia was diagnosed in women who had a tonic–clonic seizure with features of preeclampsia and no previous history of a seizure disorder such as epilepsy. Women with such history were excluded from the study. All women who were between 20 and 33^+6^ weeks’ of gestation and met the above criteria were included in the study. These data were included in a bigger study for predictive models in severe preeclampsia which included all women with severe preeclampsia from 20 weeks’ of gestation. Similar participants or part of the dataset were included in the following published works [1–4].

## Data description

A paper data collection tool was used to collect information from the labour ward delivery registers, perinatal registers and mortality registers. Data on neonatal outcome were also collected from neonatal intensive care unit and special care baby unit. Hospital case notes were retrieved and data collected regarding maternal characteristics and results of investigations. The data tool collected maternal, fetal and neonatal demographic, clinical and outcome information.

The demographic information collected included maternal age, gravidity, parity, gestational age, number of fetuses, marital status, level of education, HIV status, antiretroviral therapy, and booking status. Other data collected included aspirin therapy, past obstetric history, systolic and diastolic blood pressure, and urine dipstick proteinuria. Platelet counts, antihypertensive therapy, magnesium sulphate therapy, corticosteroid therapy and mode of delivery were other variables that were collected. Further data collected included maternal complications, fetal complications and outcomes, birth weight and adverse maternal outcomes.

The dataset contains data of 238 women who had a diagnosis of early onset severe preeclampsia/eclampsia. There were 243 babies from singleton and twin gestations. There were five sets of twins. There were 21,505 live births during the study period giving an incidence of 1.1%.

Data were manually collected from paper records and entered into the Microsoft Excel spreadsheet which can then be exported to SPSS Version 20 (IBM, Armonk, NY, USA) for analysis. Descriptive statistics can be performed and presented as frequencies and percentages for categorical variables and either mean and standard deviation or median and interquartile range depending on the way the data is distributed. Bivariate correlations of association between main independent variables and outcome measures can be performed using Pearson 2-tailed Chi square test, t-tests or Mann–Whitney U test as appropriate. The independent association of variables can be assessed using multivariable analysis. Multivariable binary logistic regression can be used to identify risk of composite adverse maternal and perinatal outcomes associated with maternal symptoms, holding other variables constant and adjusting for co-variates. Stepwise logistic regression can be calculated to develop parsimonious predictive models such as in the main protocol [3] but this time for early onset severe preeclampsia rather than the combined 2 groups of early and late onset severe preeclampsia.

The data shows the variables collected. These include maternal socio-demographic characteristics, signs and symptoms, interventions such as induction of labour and mode of delivery. It also shows the therapeutic interventions like antihypertensive treatments and corticosteroid therapy. It also contains data on maternal complications and causes of maternal deaths. Fetal characteristics include birth weight, Apgar scores and perinatal outcomes. Some of the variables have not been used in the main analysis as the programme of doctoral research evolved to have similar characteristics as in miniPIERS(Pre-eclampsia Integrated Estimate of RiSk) [5] and fullPIERS [6]. Table 1 below provides an overview of all data files/data sets described in this Data note. The Data can be accessed on Mendeley Data at http://dx.doi.org/10.17632/wrkvzf567k.2 [7].

**Table 1:** Overview of data files/data sets

Label	Name of data file/data set	File types (file extension)	Data repository and identifier (DOI or accession number)
Data File 1 [7]	Early onset preeclampsia	Excel	10.17632/wrkvzf567k.2

## Limitations

The main limitations of the dataset are that some variables are missing due to not being recorded in the case notes when the patients were seen as the data was secondary data collected retrospectively. Due to the limited resources, some variables were not done due to either the resources were not available or the emergency nature of the presentations (e.g. urine protein dipstick) left no time for them to be done. Missing variables can be dealt by multiple imputations for calculations as our preliminary analysis suggests that the data are missing at random.

## Data Availability

The data described in this Data note can be freely and openly accessed on Mendeley Data at http://dx.doi.org/10.17632/wrkvzf567k.2 [7]. Please see Table 1 and reference list for details and links to the data.

## Abbreviations

HIV: human immunodeficiency virus
MRCZ: Medical Research Council of Zimbabwe
PIERS: Pre-eclampsia Integrated Estimate of RiSk
